# Nasal Aesthetic Preferences Across Populations: A Systematic Review of Cultural, Ethnic, and Generational Trends

**DOI:** 10.1093/asj/sjag062

**Published:** 2026-03-27

**Authors:** Hamid Reza Khademi Mansour, Ali Malik, Alexander S North, Anil Joshi

## Abstract

Nasal aesthetics are central to rhinoplasty outcomes, yet ideals of beauty vary considerably across cultures, ethnicities, and generations. Current evidence is fragmented, often Eurocentric, and lacks a comprehensive synthesis. This systematic review aimed to evaluate nasal aesthetic preferences and their implications for clinical practice. The protocol was prospectively registered on PROSPERO (CRD420251009102). Systematic searches of PubMed, Embase, Web of Science, and PsycINFO were conducted from inception to June 2025. Eligible studies assessed nasal aesthetic preferences in adult (≥18 years) populations. The Newcastle–Ottawa Scale (NOS), NOS-xs, Grading of Recommendations, Assessment, Development and Evaluations, and Appraisal tool for Cross-Sectional Studies tools were used to guide critical appraisal, and findings were synthesized narratively. A total of 2881 records were screened, yielding 31 studies with 25,382 respondents. Preferences demonstrated marked regional variation: North American Caucasians favored a slight dorsal scoop with mild underprojection, whereas East Asian/Middle Eastern groups preferred a straight dorsum with more obtuse nasolabial angles. Chinese cohorts most frequently selected radix positions at the pupil, Japanese studies reported stable nasofacial angles of 30° to 33°, and Syrian findings converged around ∼31°. Generational trends indicated Millennials and Generation Z favored longer, straighter noses with augmented radix and reduced rotation. Across populations, overall facial harmony was consistently prioritized over isolated measurements. Notable contradictions and the underrepresentation of Black populations highlighted key limitations. Nasal aesthetic preferences are dynamic and context dependent. Surgeons should adopt a personalized, culturally sensitive approach to rhinoplasty, prioritizing timeless harmony over transient fashions. Future research must expand inclusivity, employ standardized measurement tools, and leverage emerging technologies to track evolving ideals and assess psychosocial outcomes.

**Level of Evidence: 3** (Therapeutic)

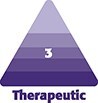

Rhinoplasty is among the most commonly performed procedures in facial plastic surgery, sought for both functional improvement and to refine nasal appearance in accordance with prevailing aesthetic ideals.^1^ Although septoplasty addresses functional deficits such as nasal obstruction, aesthetic rhinoplasty engages with more complex determinants of facial harmony, including dorsal contour, top rotation, projection, refinement, and the overall balance of the individual patient's face.^2,3^ These features are neither static nor universal; they are shaped by sociocultural contexts, reflecting variations in identity, gender expression, and regional beauty norms.^4,5^ Thus, the nose holds cosmetic and psychosocial importance, with perceived disharmony linked to reduced self-esteem, body image concerns, and, in severe cases, body dysmorphic disorder.^6-8^

The nose occupies a central anatomical position on the face, making it a key determinant of both self-perception and external judgment.^9^ Cultural norms, historical aesthetic paradigms, and media representation shape individual/collective ideals of nasal beauty. Yet these ideals are very heterogeneous: for instance, although some populations prefer a high dorsum and narrow base, others value broader bridges or softer contours. These divergent preferences are increasingly relevant in an era of globalization and ethnic diversity, where patients routinely seek procedures that enhance rather than erase ethnic features.^10-12^ As such, rhinoplasty goals should be seen not as universal standards but as personal constructs shaped by culture, age, gender, and professional insight.

Despite growing recognition of this complexity, the literature on nasal aesthetic preferences remains fragmented and disproportionately representative of Eurocentric standards.^13,14^ Although various studies have explored population-specific preferences, no comprehensive synthesis currently maps global trends. This gap may lead to clinical decisions based on limited heuristics, misaligning outcomes with patient expectations.

This systematic review addresses these gaps by synthesizing evidence on nasal aesthetic preferences across diverse populations. It examines global trends, demographic variations by ethnicity, gender, and age, and differences between layperson and expert opinions. It also considers how temporal shifts, cultural exposure, and media shape evolving ideals. Ultimately, this review aims to support culturally sensitive, patient-centered decision making in rhinoplasty by situating nasal aesthetics within broader psychosocial and demographic contexts.

## METHODS

This systematic review synthesized evidence from database inception to June 2025 and was prospectively registered with the International Prospective Register of Systematic Reviews (PROSPERO; CRD420251009102).^15^ The review methodology was developed and conducted in full accordance with the Preferred Reporting Items for Systematic Reviews and Meta-Analyses (PRISMA) guidelines to ensure transparency, methodological rigor, and reproducibility.^16^ A completed PRISMA checklist, with cross-references to corresponding sections of the manuscript, is available in Appendix A.

### Search Strategy

A systematic literature search was conducted across 4 electronic databases: PubMed (National Library of Medicine, Bethesda, MD), Embase (Elsevier, Amsterdam, the Netherlands), Web of Science (Clarivate, London, United Kingdom), and PsycINFO (American Psychological Association, Washington, DC). No restrictions were applied with respect to publication date, language, or study design, and the search strategy was tailored to each database and incorporated both Medical Subject Headings (MeSH) and relevant free-text terms to maximize sensitivity and specificity. As an example, the following search string was used in PubMed:

(Rhinoplasty [MeSH] OR Rhino OR septoplasty OR nose OR nasal) AND (Cultur* OR ethnic* OR race OR countr* OR phenotype OR nation*) AND (aesthetic OR cosmetic OR beauty OR shape OR size OR type OR angle OR projection) AND (PROMs [MeSH] OR preferences OR ideal OR standards OR likes OR dislikes)

The complete search strategies for all databases, including detailed search histories and record yields, are provided in Appendix B.

### Eligibility Criteria

Only cross-sectional surveys and observational cohort studies were included if they assessed nasal aesthetic preferences in adult patients (≥18 years), laypersons, or healthcare professionals, such as rhinoplasty surgeons. Both surgical and nonsurgical rhinoplasty studies were eligible, provided that the focus remained on aesthetic perceptions. Studies were excluded if they investigated nasal form solely in the context of congenital, posttraumatic, or functional deformities without reference to aesthetic evaluation. Editorials, letters, conference abstracts, and nonpeer-reviewed literature were not eligible for inclusion in this review.

### Study Selection

All retrieved references were exported to the Rayyan software (Cambridge, MA), where duplicate entries were automatically removed.^17^ Two reviewers (H.R.K.M. and A.M.) independently screened titles and abstracts of the remaining records using predefined eligibility criteria. Studies identified as potentially relevant underwent full-text review by the same reviewers. Any disagreements at either stage were resolved through consensus, with input by a senior reviewer (A.S.N./A.J.) when consensus could not be reached. The overall selection process was documented in a PRISMA flow diagram.

### Data Extraction

Data were independently extracted by 2 reviewers (H.R.K.M. and A.M.) using a standardized, prepiloted extraction form. Extracted variables included authorship, publication year, country of origin, study design, setting, sample size, population characteristics, funding source, and primary outcomes. Specific attention was paid to aesthetic features, such as nasal dorsum contour, tip projection, nasolabial angle, and other proportional attributes. The tools and methodologies used to capture aesthetic preferences were also recorded. Where reported, data on psychosocial correlates, cultural influences, and functional considerations were extracted. In instances of missing or unclear data, study authors were contacted for clarification. Any unresolved ambiguities or incomplete datasets were considered in the interpretation of results.

### Risk of Bias and Quality Assessment

The risk of bias for observational cohort studies was appraised using the Newcastle–Ottawa Scale (NOS), and the NOS for cross-sectional studies (NOS-xs) for cross-sectional studies.^18,19^ The certainty of evidence from observational studies was appraised using the Grading of Recommendations, Assessment, Development and Evaluations (GRADE) framework, and the certainty of evidence from cross-sectional studies was appraised using the Appraisal tool for Cross-Sectional Studies (AXIS) tool.^20,21^ The AMSTAR 2.0 tool was used to identify and address methodological deficiencies in previously published reviews on this subject.^22^

### Data Synthesis and Statistical Analysis

Data synthesis was conducted using a narrative approach because of substantial heterogeneity across the included studies. This was presented in several ways: methodological heterogeneity was prominent, with studies encompassing diverse designs, heterogeneous outcome measures (angles, ratios, linear measurements, and subjective preference rankings), and nonstandardized aesthetic assessment tools; clinical/contextual heterogeneity existed across populations; statistical heterogeneity further precluded quantitative pooling, as estimates were variably defined, inconsistently reported, and frequently not accompanied by measures of variance, rendering calculation and comparison of summary effect sizes through meta-analysis inappropriate. Consequently, the findings of this study were synthesized narratively. Qualitative results were subjected to thematic analysis, which enabled the identification of patterns and differences across demographic groups, cultural contexts, and professional perspectives.

## RESULTS

**Figure 1.**
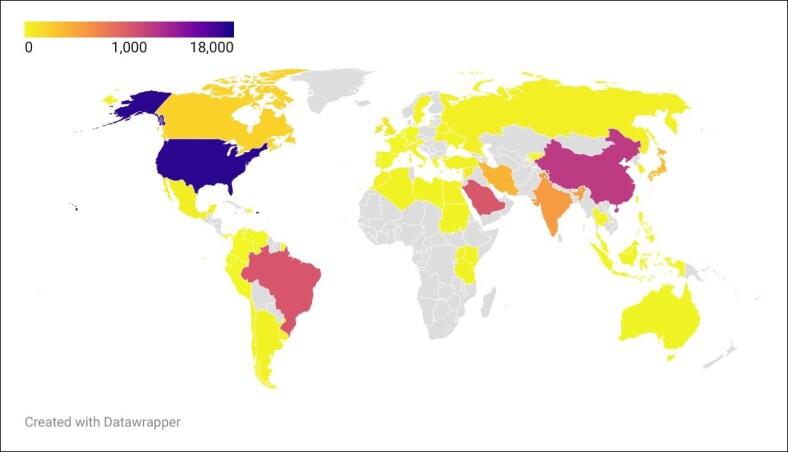
Cloropleth map showing where the included survey participants are from.

**Figure 2.**
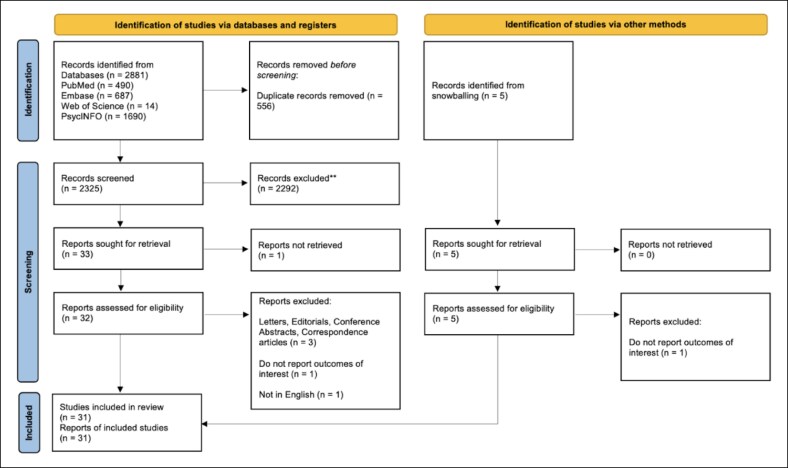
PRISMA flow diagram: illustrates the stages of article selection for this systematic review, from initial database search to final study inclusion, with numbers at each stage.

A total of 2881 records were identified through the initial database search. After removing 556 duplicates, 2325 articles remained for title/abstract screening. An additional 5 studies were identified through snowballing. Thirty-one studies met the inclusion criteria and were included in this review, representing data from over 25,382 respondents from various regions across 6 continents (Figure 1).^23-53^ None of the studies acknowledged external financial support. The selection process is shown in Figure 2; study characteristics are summarized in Appendix C.

### Nasal Dorsum and Radix

North American Caucasians preferred a slight dorsal scoop, mild over-rotation, and subtle underprojection.^49^ Chinese and Iranian groups favored a straight dorsum, sometimes with slight concavity at constant nasolabial angles.^28,34,35^ Younger North Americans (Generation Z [Gen Z], Millennials) preferred longer noses with augmented radixes and more acute nasolabial angles.^27^ European ideals emphasized higher projection and longer dorsum, whereas African and Asian cohorts favored smaller noses with lower radix; Brazilian studies also supported lower radix height as harmonious.^51,53^

In China, 8 to 10 mm nasion heights and radix positions at the pupil or eyelash line were most preferred, consistent with broader Southeast Asian preferences for elongation and higher dorsal height.^30,31,50^ Japanese participants favored stable nasofacial angles of ∼33° in men and 30° in women.^23^ Korean-American women with more acute angles were rated less attractive than those approximating Caucasian norms.^48^

Indian ideals included nasal lengths of ∼4.1 cm (female) and 4.4 cm (male), with corresponding nasofacial angles of 37.6° and 41.7°, alongside emphasis on natural outcomes and ethnic identity.^32,46^ A Syrian cohort favored 31° for both sexes.^24^ Korean-American and Iranian data suggest more obtuse nasofrontal/nasofacial angles than classical canons, with attractiveness linked to midfacial harmony (Rhee, 2017).^28, 38, 48^

### Nasal Tip Rotation

North American studies reported preferred nasolabial angles of 105° to 106° in females and ∼97° in males, although Native American, African American, and younger males often favored ∼90°.^39,40^ Preferences varied by age and ethnicity: Caucasian, Hispanic, and Millennial cohorts preferred ∼100°, whereas African American, Asian, Gen Z, and Gen X groups leaned toward ∼90°.^27^ Other large studies found female ideals of 104° to 108° without significant demographic variation.^45^ Indian-American women preferred 101.6°, more acute than Caucasians but higher than their baseline 97°.^42^

Korean-American women (92.1°) were rated most attractive when closer to Caucasian female norms (∼104°).^48^ Middle Eastern and Canadian cohorts preferred ∼97° in males and ∼110° in females, with younger respondents favoring more acute angles.^26^ Iranian women favored ∼110° and men ∼100°, although younger males preferred sharper profiles.^28,36^ Attractive Indian cohorts reported ∼104° in females and ∼101° in males (Parab et al, 2019). Syrian women after rhinoplasty showed a preference for 106° over their 101° baseline.^24^ In contrast, Middle Eastern hospital staff strongly preferred more acute values—∼90° for both sexes.^37^

In East Asia, Chinese respondents most often selected 106° (34%), with surgeons favoring it even more strongly.^30^ Japanese preferences were ∼105° in women and ∼95° in men, with variation across professions.^23^ North American surgeons generally preferred 93° to 99° for males and 95° to 100° for females, although they tended to overestimate by 2° to 3° in practice.^41,44^ McArdle et al further demonstrated that a subtle supratip break was most favored in British and Irish White females, supporting classical approaches, whereas more pronounced breaks were less desirable.^52^

### Nasal Tip Projection

North American data indicated a preference for mild underprojection combined with a scooped dorsum and overrotated tip.^49^ Using defined metrics, Crumley type 1 projection was consistently favored across age, sex, and ethnicity.^40^ In China, the most aesthetic Byrd–Hobar ratio was 0.63 (slightly higher than the classical 0.6), with younger females favoring subtle underprojection.^30^ Syrian findings similarly supported slight overprojection using both the Byrd–Hobar and Powell-modified Baum ratios.^24^ In contrast, Iranian data showed preference for 0.57 in both sexes, aligning with the classical 0.55 to 0.6 ideal.^28^ Indian cohorts reported a preferred dorsal projection of 2.08 cm in men and 1.95 cm in women, with male ideals emphasizing long, sharp, nonhumped noses with pointed rather than rounded tips.^32, 33^

### Tip Width and Alar Base

Caucasian and Asian-American women preferred narrower tips/bases.^45^ Korean-American analyses found wider roots/bases than Caucasians, with excessive narrowing producing disharmony (Rhee, 2017).^48^ Japanese and Korean studies reported a preference for narrow, straight noses with a width-to-length ratio <1, although nostril shapes differed (70% Japanese women preferred oval vs 72% Korean women triangular/teardrop).^47^

### Patients vs Professionals

Surgeons generally favor wider roots/tips and more projection than the public, with regional variation.^43^ In China, surgeons preferred straighter/smaller noses with sharper tips compared with the general population.^34,35^ Pearson and Adamson found no difference between North American patients and the general public.^49^

### Social and Economic Factors

Chinese social-media data linked rhinoplasty demand to higher-income regions, with radix/tip concerns most discussed; surgeon choice was the key decision factor.^25^ In the United States, 20% of women and 15% of men were dissatisfied with their nose; Asians were least satisfied, and Black populations were most satisfied, with no strong socioeconomic effect.^29^ Education and profession also influenced preferences: college-educated raters preferred more acute male angles.^39^ Japanese beauty professionals tolerated more obtuse female angles.^23^

### Methodological Quality and Risk of Bias

Two observational cohort studies were included in this review, and the risk of bias for these studies was appraised using the NOS tool, finding moderate-to-low risk of bias across both papers (Table 1). Twenty-one papers were appraised using the NOS-xs tool; similarly, all papers were found to have a moderate-to-low risk of bias (Appendix D). GRADE (Table 2) as well as AXIS (Appendix E) also revealed that all included studies used a robust methodology to obtain their results.

**Table 1.** Summary of Risk of Bias Appraisal Using the Newcastle–Ottawa Scale

Study (author, year)	Selection (max 4)	Comparability (max 2)	Outcome (max 3)	Total score (max 9)	Quality rating
Patel et al^42^	3	2	2	7	Moderate high
Bhat et al, 2008	2	1	3	6	Moderate

**Table 2.** Summary of Methodological Quality Appraisal Using the Grading of Recommendations, Assessment, Development, and Evaluations Framework

Study (author, year)	Outcome	Risk of bias	Inconsistency	Indirectness	Imprecision	Publication bias	Overall recommendation
Patel et al, 2012	Nasal aesthetic preferences	Low	Not serious	Not serious	Not serious	Not serious	High quality
Bhat et al, 2008	Nasal aesthetic preferences	Low–moderate	Not serious	Slight concerns	Slight concerns	Not serious	Moderate quality

### AMSTAR 2.0 Assessment

One previous systematic review was identified.^54^ In the paper by Atiyeh et al, 3 critical flaws and 2 noncritical flaws were identified using the AMSTAR 2 tool, resulting in an overall confidence rating of critically low (Table 3). The critical flaws included the absence of a priori protocol registration, lack of risk of bias assessment for included studies, and failure to explain or assess heterogeneity. The noncritical flaws related to the lack of reporting of funding sources and the limited integration of bias into interpretation. In contrast, the present systematic review addresses all of these shortcomings and, when assessed using the AMSTAR 2 tool, was rated as having high confidence. This paper reviews 11 more studies than the previously published review.

**Table 3.** Summary of Quality Appraisal Using the AMSTAR-2.0 Tool

Author	Critical flaws	Noncritical flaws	Overall confidence in results
Atiyeh et al^54^	3 (Items 1, 9, 13)	2 (Items 10, 12)	Critically low
Khademi Mansour et al (2025)	0	0	High

## DISCUSSION

To the best of the authors' knowledge, this is the largest and most methodologically robust systematic review to synthesize evidence on nasal aesthetic preferences across cultural, ethnic, and generational groups. The findings challenge the notion of fixed anthropometric ideals, demonstrating instead that aesthetic values are contextually defined. What is considered harmonious in one population may appear discordant in another, emphasizing that beauty is relational: nasal proportions derive their appeal in interaction with overall facial balance and within the cultural frameworks through which they are interpreted.

### Cultural and Generational Meanings

The review confirms that cultural and ethnic background exerts a profound influence on nasal aesthetic preferences. Notably, however, Korean-American populations demonstrated that attractiveness is judged not solely by ethnic morphology but also by prevailing cultural reference points. In this cohort, nasal shapes closer to Caucasian norms were rated as more attractive despite differing baseline features. This highlights the complex interplay between ethnicity and sociocultural environment, suggesting that aesthetic ideals among ethnic minority populations are shaped both by inherited morphology and by external cultural pressures, particularly Western beauty standards. Importantly, this finding underscores that data derived from native populations cannot be directly extrapolated to diaspora or minority groups living in different cultural contexts. Future research focusing on ethnic populations within single host countries may therefore provide the most meaningful insights, as it would account for the layered influence of both ethnic origin and the surrounding cultural environment on aesthetic preferences.

Generational trends add further complexity. Contemporary cohorts, particularly Millennials and Gen Z, favor longer, straighter noses with augmented radix and reduced tip rotation, diverging from the highly rotated “ski-slope” nose that dominated previous decades. These shifts reflect broader societal movements privileging authenticity and naturalism in beauty standards.^55,56^ Importantly, social media plays a critical role in accelerating these generational changes. Platforms such as Instagram and TikTok circulate curated images and before-and-after transformations that shape expectations and rapidly disseminate aesthetic ideals.^57^ Although such exposure promotes diversity in beauty norms, it also accelerates their volatility.^58^

A counterpoint, however, is that the homogenizing effect of globalized social media may gradually erode the influence of ethnicity, as younger patients increasingly aspire to similar ideals regardless of their background. Yet, anatomical realities such as variation in skin thickness, cartilage integrity, and skeletal support across ethnic groups mean that identical outcomes cannot be universally achieved. For clinicians, the central challenge lies in reconciling the permanence of surgical change with the transience of digital trends, highlighting the importance of counseling younger patients about timeless harmony over fleeting fashions.

### Critical Insights

Analysis of tip rotation and projection revealed both inconsistencies and contradictions. Female ideals typically clustered between 104° and 110°, whereas male ideals fell closer to 95° to 100°, although extremes spanned from <90° to >110° depending on subgroup and methodology. Projection was generally favored as mild underprojection in North America, slight overprojection in China and Syria, and closer to classical values in Iran. Harmony, rather than any single measurement, emerged as the most consistent determinant of attractiveness. Contradictory findings, such as those reported by Alshawaf^26^ and Alharethy, underscore how methodology, sampling, and subcultural variation shape results, undermining attempts to universalize aesthetic “truths.”^37^

### Clinical Implications

For clinical practice, these findings reinforce the need for personalization and cultural competence. Surgeons must approach rhinoplasty consultations without preconceived ideals, engaging in dialogue to elicit each patient's aesthetic vision. Ethnicity, cultural background, and generational identity may inform preferences, but stereotyping must be avoided; not all members of a group desire the same nasal form. Effective, patient-centered communication, such as exploring the cultural or familial significance of nasal features or discussing admired role models, builds trust and ensures alignment between surgical planning and patient values.

In practical terms, surgical planning should integrate anatomy, preference, and identity. Digital morphing tools can not only help reconcile differences between surgeon and patient perceptions, but surgeons must also educate patients regarding anatomical limitations and the risks of pursuing extreme or transient ideals.^59^ By prioritizing timeless harmony over fashion, personalized and culturally sensitive rhinoplasty can improve satisfaction rates and psychological outcomes.^60^

### Methodological Strengths and Limitations

This review benefited from studies employing standardized photographic manipulations, large cross-cultural surveys, and the inclusion of both lay and expert perspectives. Such designs enhanced validity and provided broad insight into plural aesthetic ideals.

However, several limitations must be acknowledged. First, the included studies employed heterogeneous metrics to assess aesthetic preferences, ranging from anthropometric ratios and angles to subjective surveys. This methodological diversity complicates interpretation and limits direct comparability. Even when single features such as the nasolabial angle were manipulated, participants invariably judged the overall facial profile rather than the isolated parameter. Consequently, it is difficult to fully disentangle the effect of specific nasal attributes from the broader harmony of the face.

Secondly, the studies span more than 2 decades. Given the well-documented generational shifts in beauty ideals, comparing results across time is problematic; preferences expressed in older datasets may no longer reflect contemporary norms, particularly in the era of social media.^61^ A further limitation is that high-quality evidence is disproportionately concentrated in certain regions, such as the United States. In contrast, robust data from other parts of the world remain limited, restricting the ability to make meaningful global comparisons. This geographic imbalance highlights the need for further studies in underrepresented populations to provide a more comprehensive understanding of cultural and ethnic variation in nasal aesthetic preferences. Next, many studies used digitally altered photographs of unrelated models. Although this standardizes stimuli, such images may not capture what individuals would prefer for themselves. Nasal harmony depends on unique facial structures, and preferences elicited in isolation may not translate to personal aesthetic choices. Finally, representation across populations remains uneven. Although East Asian and Middle Eastern groups are relatively well studied, Black populations are almost entirely absent from the literature. This gap perpetuates Eurocentric bias, narrows the generalizability of findings, and reinforces the need for more inclusive research designs.

### Directions for Future Research

Future work should prioritize inclusivity, standardization, and methodological innovation. Black, Indigenous, and mixed-ethnicity cohorts remain underrepresented, as do intra-ethnic variations within large regions such as Latin America and Africa. Standardized, validated instruments for measuring aesthetic preferences are urgently required to overcome current methodological fragmentation. Emerging technologies, 3-dimensional morphing, virtual reality, and machine learning applied to large image datasets, offer opportunities for more reproducible and culturally adaptable assessments.^62^

Longitudinal research is also needed to capture generational change, disentangling enduring ideals from transient fashions. Beyond aesthetics, future studies should examine psychosocial outcomes, including self-esteem, cultural identity, and long-term satisfaction when results align with patient preferences. Finally, rigorous evaluation of the impact of digital and social media on aesthetic norms is essential, given its pervasive influence on younger cohorts.

## CONCLUSIONS

This review demonstrates that nasal aesthetics are dynamic, context-dependent constructs shaped by cultural background, generational trends, and social influences rather than fixed anthropometric ideals. Preferences for dorsum shape, radix position, tip rotation, and projection varied widely across populations, with social media accelerating shifts among younger cohorts. Clinically, these findings reinforce the need for personalized, culturally competent rhinoplasty that prioritizes timeless harmony and aligns with individual identity while avoiding the imposition of universalized norms.

Future research must address persistent gaps by including underrepresented populations, particularly Black, Indigenous, and mixed-ethnicity groups, and by adopting standardized and validated methods to measure preferences. Incorporating technologies, such as 3D morphing and machine learning, alongside longitudinal designs, could better capture evolving ideals and link them to psychosocial outcomes such as satisfaction and cultural identity. In recognizing nasal aesthetics as relational and evolving, this review provides a framework for advancing both scholarly understanding and patient-centered surgical practice.

## Supplemental Material

This article contains supplemental material located online at https://doi.org/10.1093/asj/sjag062.

## Supplementary Material

sjag062_Supplementary_Data
