# Supplementary material for: Nasal Aesthetic Preferences Across Populations: A Systematic Review of Cultural, Ethnic, and Generational Trends
Source: Aesthet Surg J. 2026 Mar 27;46(8):NP73–80. doi: 10.1093/asj/sjag062 (PMC13458594; doi:10.1093/asj/sjag062)
Supplement: Supplementary file 1 [file sjag062_supplementary_data.zip › Appendix B.docx]

**Appendix B**. Search Strategy

(Rhinoplasty [mesh] OR rhino* OR septoplasty OR nose OR nasal) AND (cultur* OR ethnic* OR race OR countr* OR phenotype OR nation*) AND (aesthetic OR cosmetic OR beauty OR shape OR size OR type OR angle OR projection) AND (PROMs [mesh] OR preferences OR ideal OR standards OR likes OR dislikes)

Total Results: 2881

Pubmed: 490

Embase: 687

PsycINFO: 1690

Web of Science: 14
