# Supplementary material for: Nasal Aesthetic Preferences Across Populations: A Systematic Review of Cultural, Ethnic, and Generational Trends
Source: Aesthet Surg J. 2026 Mar 27;46(8):NP73–80. doi: 10.1093/asj/sjag062 (PMC13458594; doi:10.1093/asj/sjag062)
Supplement: Supplementary file 1 [file sjag062_supplementary_data.zip › Appendix C.docx]

**Appendix C**. Study Characteristics

| Author, Year, Country | Study Design | Population, Sample size | Outcomes Assessed | Preferred features | Sex/Regional Differences | Key Findings |
| --- | --- | --- | --- | --- | --- | --- |
| Okumura et al., 2025, Japan | Cross Sectional Survey | Japanese men and women, aged 20–59, 360 | Nasal tip projection, nasofrontal angle, nasal dorsum shape, nasolabial angle, radix height | Straight dorsum, tip projection aligned with lower border of upper lip, nasolabial angle 95–105° | Female participants preferred slightly higher nasal tip projection and radix than males; age and sex both influenced preferences | Japanese aesthetic preferences prioritize a straight dorsum and subtle, natural tip projection; notable influence of demographic variables on preference patterns |
| Torbey et al., 2024, Syria | Cross Sectional Study | Medical students from Syrian Private University; 47% males, 53% females; mean age 22.25, 100 | Nasofrontal angle, nasolabial angle, nasomental angle, nasofacial angle, nasal index, tip projection ratios (Byrd/Hobar, Powell/Baum) | Rhinoplasty-negative group preferred more acute nasofrontal angles (144.59°), wider nasolabial angles (106.32°), and narrower nasal index (62.91) | No specific sex breakdown in preferences; some demographic distinctions between rhinoplasty-positive vs. negative groups noted | Syrian students preferred narrower nasal features, more acute angles than other populations; trends aligned with Syrian surgical norms; regional aesthetic preferences closer to Turkish and Saudi populations, differing from Iranian and Western preferences; post-op satisfaction noted in rhinoplasty-positive group reflected minimal preference for change. |
| Liu et al., 2024, China | Cross Sectional Analysis | Chinese social media users interested in rhinoplasty, mostly female, aged 18–35, across all 34 provinces,  1,065 posts, 102,153 comments, 239,383 collections, 640,579 likes | Nasal tip, nasion, dorsum shape, materials used (e.g., hyaluronic acid), sentiment/motivation analysis | Natural-looking, harmonious noses; nasal tip most discussed (37.3%); non-surgical procedures using hyaluronic acid popular | Higher demand and engagement in southern provinces; interest correlated with economic prosperity; minimal northern engagement | Cultural preference for subtle, harmonious nasal features over exaggerated ones; high use of hyaluronic acid for non-surgical rhinoplasty; regional trends linked to socioeconomic factors; fear of complications common concern; southern China showed higher demand and favored procedures for broader, lower nasal traits; affordability and fear of surgery drive non-invasive preferences |
| Al-Shawaf et al., 2024, Saudi arabia | Cross Sectional Study | Participants aged 20–39. Predominantly female (81.2%). National distribution: Canada 30.5%, Kuwait 30.5%, Saudi Arabia 19.8%, Bahrain 10.2%, Lebanon 2.5%, others 6.6%. Majority were students (49.7%) or employees (34%), 197 | Ideal nasolabial angle (NLA) for male and female noses; demographic influences | Male NLA: 90°–95° preferred (mean 97.1°); Female NLA: 110°–115° preferred (mean 109.5°) | Younger participants preferred more acute NLAs; students preferred 100°, employees preferred 95°; no major sex or national differences | Cultural variation observed: Middle Eastern populations favored slightly more obtuse NLAs vs. Canadians. Aesthetic preferences appear consistent across nationalities despite regional variation. Use of Caucasian-based 3D models limits generalizability. Personal or family history of cosmetic surgery did not significantly alter preferences. Highlights the need for ethnically tailored rhinoplasty planning. |
| Patel et al., 2023, USA | Cross Sectional Survey | Female volunteers aged 18–55 from the general population. Grouped by generation: Gen Z (18–23), Millennials (24–39, split into 20s and 30s), Gen X (40–55), 177 | Nasal radix height and nasolabial angle (90°, 100°, 110°) | Gen Z, Millennial 20s/30s preferred a more augmented nasal radix (+5 mm); Gen X preferred baseline radix. NLA: Gen Z, Millennial 20s, and Gen X preferred 90°, while Millennial 30s preferred 100°. | African Americans and Asians preferred more natural radix height and a more acute NLA (90°); Caucasians and Hispanics leaned toward 100° NLA. | Younger generations favored longer noses and less rotated tips, reflecting non-surgical rhinoplasty trends. Preferences varied significantly across ethnicities (p < 0.001). Cultural and social influences strongly impacted aesthetic ideals. Study aligns with broader shifts toward natural nasal profiles and minimally invasive procedures. |
| Tavakoli et al., 2023, Iran | Cross Sectional Study | Predominantly Iranian participants. 79.8% female, mean age = 31.2 years. Recruited from 35 cities, mostly Tehran. 59.6% had prior aesthetic surgery, 203 | Nasofrontal angle (NFA), nasolabial angle (NLA), dorsal height, alar width, tip projection | Females preferred NFA ~135°, NLA = 110°, and narrow alar width (−2 mm). Males preferred NFA = 140°, NLA = 100°, and alar width equal to intercanthal distance. Both preferred a straight dorsum and TP = 0.57. | Males preferred more acute female NLA; females preferred wider NLAs. Younger adults favored more acute angles. Surgery history and city influenced preferences. | Iranian participants showed culturally distinct preferences, including wider NFAs and thinner female noses. Preferences were shaped by gender, age, and history of surgery. Compared to Western ideals, Iranians favored more angular, straight nasal features. Women aligned more with neoclassical ideals than men. |
| Frederick et al., 2022, USA | Cross Sectional Survey | U.S. adults aged 18–65. Ethnically diverse: White (75.2%), Black (6.7%), Hispanic (4%), Asian (6.1%), Native American (0.5%), and mixed-race participant, 11,620 | Nose satisfaction as part of facial image satisfaction | Not explicitly stated; assessed satisfaction levels rather than ideal feature preferences. | Asian participants reported the lowest nose satisfaction. Women reported lower satisfaction than men. Gay/bisexual men had more dissatisfaction than heterosexual men. | Asian participants had the lowest satisfaction with nose and facial appearance; cultural pressure and Western beauty ideals impacted self-perception. Black participants had higher satisfaction than White peers. BMI and relationship status influenced overall facial satisfaction. The study highlighted the impact of race, gender, and sexuality on nasal and facial aesthetics perception. Focus was on dissatisfaction rather than ideal features. |
| Sun et al., 2022, China | Cross Sectional Survey | 653 laypeople and 50 plastic surgeons. Participants from both developed (41.5%) and developing (58.5%) Chinese regions. 16.6% reported personal history of cosmetic surgery. | Nasal tip projection (NTP) to nasal dorsum length (NDL) ratio; nasolabial angle (NLA) | Ideal NTP/NDL ratio: 0.63. Ideal NLA: 106°. | Preferences were consistent across sex, age, and region. Plastic surgeons had stronger consensus on ideal features than laypeople. | Chinese respondents preferred more subtle nasal projection than Western norms. 0.63 NTP/NDL ratio was most popular across all groups, including 66% of plastic surgeons. Ideal NLA (106°) was preferred uniformly. Cultural norms influenced preferences, with a tendency to maintain ethnic characteristics despite desire for augmentation. Regional and surgical history did not significantly impact preferences. |
| Liang et al., 2022, China | Cross Sectional Survey | (59.3% women; 9.5% plastic surgeons); mostly aged 18–35. Predominantly Han Chinese with 8.2% non-Han. Included laypersons and individuals with or without prior cosmetic surgery, 777 | Nasion height, nasion position, forehead height | Nasion height: 8–10 mm; Nasion position: center of pupil; Forehead height: 4 mm above nasion | Non-Han participants preferred pupil-level nasion more than Han Chinese (60% vs 41.9%). Plastic surgeons had more consistent preferences. | Chinese participants preferred lower nasion heights and centered positions, aligning with local aesthetic norms. Surgeons and patients with cosmetic surgery history leaned toward subtler enhancements. Preferences differed by ethnicity, highlighting the need to tailor rhinoplasty planning to cultural and anatomical expectations. Plastic surgeons emphasized subtlety to retain ethnic identity and facial harmony. |
| Corduff et al., 2020, Singapore | Cross Sectional Survey | Asian patients seeking aesthetic treatment (assessed by aesthetic physicians), 68 | Nose length, dorsal height, nasofrontal angle, nasolabial angle, subnasale projection, midface projection, lower face and chin shape, lip-to-lower-face ratio | Preferred features included elongated noses with higher dorsal height, balanced nasolabial angles, projected midface, V- or tapered-U lower face, 1:2 lip ratio, and oval/rounded upper face | Regional and sex differences: APAC female patients requested more rounded upper faces and pointed chins (esp. in Taiwan, Singapore, Thailand); males often preferred square/curved chins and flat brows; Singaporean, Thai, Korean women preferred arched brows, but Thai/Korean women often wanted flat brows; most male patients preferred horizontal eye cants, women varied by country | Asian aesthetic preferences emphasize nose elongation and higher dorsal height, narrower lower face (V/tapered), projected midface, and balanced lip proportions; consensus highlighted wide variability across APAC regions, showing cultural and sex-related diversity in nasal/facial ideals:contentReference[oaicite:0]{index=0} |
| Parab et al., 2019, India | Cross Sectional Study | young Indian adults (18–25 years), 110 males and 111 females, representing North, South, East, and West India. No prior nasal surgery, trauma, or deformities. | Nasal length, width, nasolabial angle, nasofrontal angle, nasofacial angle, nasomental angle | Nasal shapes proportionate to broader Indian facial features; no universal "ideal" stated | Males had longer, wider noses with smaller nasolabial and nasofrontal angles than females | Indian noses were shorter and wider than European and Chinese populations. Nasal angles smaller than in Caucasians but greater than in Afro-Americans. Despite regional diversity, no significant regional differences in nasal parameters were found. Cultural emphasis on natural outcomes and maintaining ethnic identity. First study proposing average aesthetic nasal parameters specific to the Indian population, supporting ethnic-specific rhinoplasty planning. |
| Balaji et al., 2019, India | Cross Sectional Study | 27 male patients aged 22–42 years seeking facial aesthetic surgery. Ethnic groups: 15 Australoid (Indian), 6 Mongoloid/Southeast Asian, 3 Black, 3 Caucasian. | Nasal length, dorsum shape, nasal tip projection, nares width | Long, straight, sharp noses with pointed tips; wide nares with adequate projection | Indian males desired “masculine” sharp noses; Mongoloid patients emphasized subtlety and harmony; Black patients wanted narrower nasal bases while preserving identity | Cultural and psychosocial factors influenced aesthetic preferences. Indian males sought dominant, sharp features driven by Bollywood/media ideals. Ethnic-specific trends noted: Southeast Asians preferred subtle corrections; Black participants emphasized identity preservation; Caucasians requested minimal adjustments. Surgeons are encouraged to adopt culturally sensitive, individualized approaches for optimal aesthetic satisfaction. |
| Samizadeh et al., 2018, China | Cross Sectional Survey | 1,417 participants (599 men, 818 women); majority aged 25–35 years (avg. age 33.5); 90% had university education; represented 32 provinces including Shanghai, Beijing, Guangdong | Nose shape, facial profile preferences, willingness for cosmetic surgery | Straight nasal profile (42.27%) most preferred; concave profile second (29.64%); pointed tip also valued (10.73%) | No sex-specific differences reported; urban regions (e.g., Shanghai) overrepresented, suggesting regional trend bias | Participants favored ethnic-specific traits over Westernized features; strong preference for straight/conservative profiles and non-surgical procedures (82.22%); 83% believed attractiveness impacted social/professional success; high dorsum and defined tip preferred; aesthetic ideals driven by harmony and natural appearance rather than exaggeration. |
| Samizadeh et al., 2018, China | Cross Sectional Survey | 596 aesthetic practitioners (505 women, 69 men); aged primarily 18–30; 78.2% university-educated; included cosmetic doctors, plastic surgeons, dermatologists, beauty consultants | Preferences for nasal profiles, tip projection, bridge height; facial harmony | Straight nasal profile with a well-projected tip (Sketch 7) preferred by 60.8%; slightly concave also preferred (22.2%) | No significant sex-specific analysis reported; practitioners leaned toward sharper, more refined nasal features than laypersons | Practitioners preferred more defined nasal features than laypersons, aligning with Korean-influenced ideals of straight dorsum and sharp tip; aquiline/downturned noses least preferred; cultural influence noted in emphasis on facial harmony and refinement; highlights divergence between professional and public perceptions and reinforces need for tailored aesthetic consultations respecting ethnic identity and patient goals. |
| Mafi et al., 2018, Iran | Cross Sectional Study | Iranian adults (16–40 yrs),  200 (100M, 100F) | Nasal tip angle, lip projection, facial proportions | Fuller lips, balanced vertical proportions | Iranian participants showed different aesthetic preferences compared to North American Caucasians; no sex-specific differences reported | Cultural factors influenced profile preferences; lacked clear focus on nasal feature quantification; weak comparative analysis |
| Saad et al., 2018, Spain | Cross Sectional Study | 147 beauty pageant contestants (female) representing 7 ethnic groups (Mediterranean, Eastern Europe, Scandinavia, Southwestern Europe, East Africa, China, Japan),  147 (21 per group, except China n=16, Japan n=20) | Nasal dorsum length, tip projection, radix position, nasolabial angle, intercanthal distance, alar base width, mouth width, facial thirds | European/Mediterranean groups: longer dorsum, higher tip projection, radix at lash line–crease, nasolabial angle ~97–99°; East Africans/Asians: shorter dorsum, smaller noses, radix lower (pupil–lash line), nasolabial angle more acute (East Africans ~87°) | Only female contestants studied; variations across ethnic groups but not within sexes; regional distinctions more pronounced than sex-based ones | Study showed NCCs and classical aesthetic standards did not apply across ethnicities; significant interethnic variability in nasal proportions; Mediterranean/European ideals favored higher projection and longer dorsum, whereas African/Asian groups had smaller noses with lower radix. |
| Al-harethy et al., 2017, Saudi Arabia | Cross Sectional Study | 1,027 Middle Eastern participants (506 males, 521 females); aged ~22; included Egyptians, Syrians, Tunisians, Yemenis; mostly medical students, nurses, hospital staff | Nasolabial angle (NLA) preferences for male and female profiles | Males: 89.39° ± 3.66°; Females: 90.62° ± 5.15°; preferred more acute angles than Western standards | Minimal sex differences; both male and female raters showed preference for acute NLA in both sexes | Middle Eastern participants preferred less rotated nasal tips compared to Western ideals; acute NLAs favored by both sexes; cultural norms play a strong role; emphasizes the need to tailor rhinoplasty planning to local aesthetic preferences; Western norms (NLA 95°–115° for females) may not be universally applicable. |
| Rhee et al., 2017, South Korea | Cross Sectional Survey | Composite images from 44 Asian female celebrities (Chinese, Japanese, Korean) and 20 attractive Caucasian women (online-sourced images) | Nasal width/height ratio, nasolabial angle, nasofrontal angle, nasal depth | Asians: wider noses (72.4%), more obtuse NLA (109.5°), greater nasofrontal angle (141.5°); Caucasians: narrower noses (69.3%), NLA = 101.8°, nasofrontal angle = 137.9° | No sex-specific data reported; compared Asian vs. Caucasian morphotypes | Asian preferences favored softer, wider nasal features with more obtuse angles; Caucasian preferences leaned toward narrower, elongated noses with more acute angles; globalization trends showed convergence in younger populations; cultural ideals emphasized harmony in Asians vs. structural definition in Caucasians. Study reinforces ethnic-specific norms for aesthetic planning. |
| Sinno et al., 2014, Canada | Cross Sectional Study | 98 participants aged 18–70; Caucasian: 62.7%, Asian: 18.6%, Black: 3.9%, Native American: 0.9%, Mixed/Other: 9.8% | Preferred nasolabial angles for males (90°, 100°, 105°) and females (100°, 105°, 110°); demographic influences | Females: Most preferred NLA = 105° (mean 104.9° ± 4.0); Males: Most preferred NLA = 90° (mean 97.0° ± 6.3); acute angles favored for males, intermediate for females | Ethnic: African Americans and Native Americans strongly preferred 90° for males; Caucasians and Asians showed varied but similar tendencies. Age: >50 preferred more obtuse NLAs; <30 preferred acute NLAs | Male NLA preferences were more affected by race, age, and education than female ones; younger participants and certain ethnic groups (AA, NA) favored acute angles for men; minimal variation in female nasal preferences. Results emphasize the value of demographic tailoring in rhinoplasty planning. |
| Ahmed et al., 2014, USA | Cross Sectional Survey | Young White women (18–25 years); focus group (n=106, UC Irvine students) and online participants (n=3,872 via social media) | Ideal nasal tip projection (NTP) and rotation via Crumley 1, Crumley 2, and Goode methods; preferences for tip rotation angles (96°–116°) | 106° tip rotation most preferred across all methods; Crumley 1 was the most aesthetically preferred NTP method | Study population restricted to young White women; no ethnic or sex comparisons; greater preference consistency among women rated as more attractive | 106° columellar facial angle rated most aesthetic; Crumley 1 method preferred especially for highly attractive faces; nasal tip projection and rotation were interdependent; provides evidence-based insights for rhinoplasty planning; not generalizable to other ethnicities |
| Brown et al., 2013, USA | Cross Sectional Study | 20 rhinoplasty patients (10 male, 10 female) from various ethnic backgrounds; evaluated by 28 rhinoplasty surgeons (71% North American, 29% international) | Ideal nasolabial angle (NLA); expert vs non-expert preference comparison; surgeon overestimation tendencies | Male: 95.6° ± 2.7° (95% CI: 93.9°–97.3°); Female: 98.5° ± 2.6° (95% CI: 96.8°–100.2°) | Female NLA wider than male NLA (statistically significant); consistent expert preferences across regions | Experts preferred narrower NLAs than prior guidelines (90°–120°); experts overestimated NLA by 2.7° on average; emphasis on proportionate, natural aesthetics over rigid neoclassical norms; results suggest convergence toward universal aesthetic standards among surgeons |
| Patel et al., 2012, USA | Retrospective Observational Study | 15 Indian American women dissatisfied with their noses (prospective); 20 Indian American rhinoplasty patients (retrospective; mixed gender) | Nasolabial angle, dorsal profile, nasal width, tip projection; cultural influences | Avg NLA: 97.4°, desired 101.6° (vs. 104.2° in White women); 67% wanted dorsal reduction, 80% sought width narrowing, 40% wanted tip projection ↑, 33% ↓ | Indian Americans preferred modest changes and natural harmony; emphasis on preserving ethnic identity | Cultural value placed on subtle refinement over dramatic alteration; Bollywood and Western media influence growing; three nasal morphology categories described with tailored operative strategies; conservative techniques used to preserve South Asian ethnic features |
| Broer et al., 2012, USA | Cross Sectional Survey | 1,226 respondents from 50 countries: plastic surgeons (n=720), general public (n=506); majority Caucasian (71%), Hispanic (15%), East Asian (8%); age 18–87; 61% male. Regions included North America, Latin America, Europe, Asia, Oceania, North Africa. | Nasal root width, tip width and projection, dorsum shape, nasolabial angle, columellar show, chin/lip projection | Surgeons preferred wider nasal roots and tips; Latin American surgeons and public preferred narrower noses and projecting tips; North Americans preferred higher dorsum projection; East Asians preferred wider nasal bases and tips; Caucasians preferred balanced proportions | Male respondents (public and surgeons) preferred broader nasal features; females leaned towards narrower, refined shapes. Latin American public favored pronounced projection; East Asians preferred wider features; surgeons’ preferences differed from public’s, favoring structural harmony | Strong regional and ethnic variation in nasal preferences; surgeons emphasized facial harmony while public preferences were more culture-driven. Latin American and East Asian respondents showed distinct aesthetic ideals. Public and surgeons differed notably in ideal nasal dimensions and projections. |
| Armijo et al., 2012, USA | Cross Sectional Study | 20 rhinoplasty patients (10 men, 10 women) of diverse ethnic backgrounds; evaluated by 16 professionals (surgeons, nurses, staff); mean age ~38 years. | Nasolabial angle in males and females | Males: 95.96° ± 2.57° (range: 93.4°–98.5°); Females: 97.79° ± 2.32° (range: 95.5°–100.1°); both narrower than traditional 90°–120° range. | Surgeons preferred more acute angles than laypeople; lay evaluators showed more variability in preferences. | Narrowed the ideal nasolabial angle range compared to historical standards; highlighted shift from Westernized obtuse angles to more acute ones across diverse ethnicities. Surgeons favored slightly sharper angles than non-experts. Ethnic-specific differences not analyzed despite multiracial sample. Suggests individualized planning is critical in rhinoplasty. Limitations include small image sample and lack of ethnic subgroup analysis. |
| McArdle et al., 2012, UK | Cross Sectional Survey | 100 participants (50 male, 50 female; nurses, doctors, medical students, laypersons; mostly White, some Asian, Hispanic, African-American; mean age ~29 years) | Supratip break (STB) angulation—subtle vs. marked | Subtle STB angulation preferred (63–64% overall preference); subtle = most aesthetically pleasing | Little sex difference: both male (60.6%) and female (63.4%) groups significantly preferred subtle STB; cross-ethnic participant mix showed consistent trend | Study provides strong evidence that subtle supratip break is most favored in White female nasal aesthetics; supports classical surgical approaches emphasizing slight tip break; marked STB less desirable |
| Gomes et al., 2011, Brazil | Cross Sectional Survey | Three groups: 20 healthcare professionals (ENT, plastic, orthodontics, maxillofacial), 20 fine artists, 120 laypersons (60M, 60F; aged 18–30; all with ≥undergraduate education) | Nasal radix height (low = pupil level, regular = tarsal fold, high = above eyelid crease) | Lower radix height (at pupil level) considered most aesthetically pleasing; high radix (Greek/classical) considered least attractive | No significant sex or age differences; higher education associated with stronger preference for low radix height | Brazilian aesthetic preferences differ from North American/European standards; lower nasal radix height judged most harmonious; cultural factors strongly shape nasal aesthetics. |
| Biller et al., 2009, USA | Cross Sectional Survey | General public including physicians, nurses, students, and laypeople (n=171); represented ethnicities: White (48.5%), Asian (32.2%), Hispanic, African American, East Indian, Middle Eastern, Multiethnic | Nasolabial angle (NLA), nasal tip width | Narrow tip widths (35% of alar base); moderate NLA (104°–108° for younger, up to 116° for older); more conservative features preferred by older participants | Asians showed slightly higher tolerance for wider tips; women preferred lateral features, men preferred medial; age also influenced preferences | Extreme NLAs (96°, 116°) considered least attractive; thin nasal tips generally favored; aesthetic preferences shaped more by age and facial proportions than ethnicity; strong age and sex influences on aesthetic ideals |
| Bhat et al., 2008, India | Retrospective Observational Study | 316 Indian patients (192 females, 124 males), aged 17–35 years; primarily from Mumbai but included other Indian regions; 15-year period | Common nasal deformities, aesthetic goals, techniques, satisfaction | Moderate projection, straight dorsum, narrower nasal pyramid and alar base; cartilage grafts widely used | Younger patients (17–25 years) most likely to seek surgery; broad tips and bases common across sexes | Ill-defined nose was the most frequent deformity (46.8%). Augmentation (65.8%) and narrowing procedures (alar wedge resection in 55.7%, osteotomies in 69.3%) were common. Strong preference for preserving ethnic identity. Patient satisfaction was high (80.7% rated outcome as good/excellent). Emphasizes tailored surgical approaches (e.g., stacked cartilage grafts) for Indian anatomy. |
| Dobke et al., 2006. USA | Cross Sectional Survey | 100 East Asian women (50 Korean, 50 Japanese) aged 18–50, living in Southern California; matched for age and lifestyle; no history of plastic surgery | Nasal width/length ratio, dorsum contour, nostril shape, overall nose size | Both groups preferred straight, narrow noses with width/length ratio <1; Koreans preferred triangular nostrils and fuller lips; Japanese preferred oval nostrils and smaller, delicate features | Koreans favored larger noses and stronger facial features; Japanese participants preferred more delicate, harmonized features | 92% of Koreans and 100% of Japanese preferred straight, narrow noses. Korean women showed a stronger preference for teardrop-shaped nostrils and fuller facial features; Japanese preferred oval nostrils and natural aesthetic harmony. Cultural ideals influenced distinct nasal preferences. Oval face shape was universally preferred. |
| Choe et al., 2004, USA | Cross Sectional Study | 72 Korean American (KA) women aged 18–35, evaluated by 10 KA judges (5 male, 5 female); 90% born in South Korea, 10% in the U.S. | Nasal width, root width, columella length, nasal length, nasolabial angle | Most attractive KA women had nasal features closer to North American White (NAW) norms—narrower base/root, more obtuse NLA | NAW women had narrower nasal bases and more obtuse NLAs; KA women had more acute NLAs (92.1° vs. 104.2°), broader nasal roots and bases | KA women had broader noses and more acute nasolabial angles than NAW women. However, the most attractive KA women (top 10%) had nasal traits resembling NAW norms, suggesting assimilation of Western ideals. Preference was for refinement without erasing ethnic identity. Surgeons should avoid over-Westernizing Asian noses. |
| Pearson et al., 2004, USA | Cross Sectional Study | 42 participants (27 rhinoplasty patients + 15 general public). White women evaluated via composite images. | Nasal profile preferences: dorsal scoop vs. hump, tip rotation, projection; comparison between rhinoplasty patients and general public. | Both groups preferred a slight dorsal scoop, mild tip rotation, and under-projection. No major differences between patients and public. | Study focused only on White women; no differences found by demographic. Preferences aligned with Western ideals. | Rhinoplasty patients and the general public shared similar nasal aesthetic ideals. Under-projection and scooped dorsum preferred. Surgeons should not assume unrealistic expectations; aim for natural results. |
