# Supplementary material for: Nasal Aesthetic Preferences Across Populations: A Systematic Review of Cultural, Ethnic, and Generational Trends
Source: Aesthet Surg J. 2026 Mar 27;46(8):NP73–80. doi: 10.1093/asj/sjag062 (PMC13458594; doi:10.1093/asj/sjag062)
Supplement: Supplementary file 1 [file sjag062_supplementary_data.zip › Appendix D.docx]

**Appendix D**. Summary of Risk of Bias Appraisal Using the Newcastle-Ottawa Scale for Cross-sectional Studies (NOS-xs)

| Study (Author, Year) | Selection (max 2) | Assessment of Exposure/Outcome (max 4) | Confounding Factors (max 3) | Total Score (max 9) | Quality Rating |
| --- | --- | --- | --- | --- | --- |
| Okumura et al., 2025 | 2 | 4 | 2 | 8 | High |
| Torbey et al., 2024 | 2 | 4 | 2 | 8 | High |
| Liu et al., 2024 | 2 | 4 | 2 | 8 | High |
| Al-Shawaf et al., 2024 | 2 | 4 | 2 | 8 | High |
| Patel et al., 2023, USA | 2 | 3 | 3 | 8 | High |
| Tavakoli et al., 2023, Iran | 2 | 2 | 2 | 6 | Moderate |
| Frederick et al., 2022, USA | 2 | 4 | 3 | 9 | High |
| Sun et al., 2022, China | 2 | 4 | 2 | 8 | High |
| Liang et al., 2022, China | 2 | 4 | 2 | 8 | High |
| Corduff et al., 2020, Singapore | 2 | 3 | 2 | 7 | Moderate–High |
| Parab et al., 2019, India | 2 | 2 | 2 | 6 | Moderate |
| Balaji et al., 2019, India | 1 | 3 | 3 | 7 | Moderate–High |
| Samizadeh et al., 2018, China | 2 | 3 | 2 | 7 | Moderate–High |
| Samizadeh et al., 2018, China | 2 | 3 | 2 | 7 | Moderate–High |
| Mafi et al., 2018, Iran | 2 | 3 | 2 | 7 | Moderate–High |
| Saad et al., 2018, Spain | 2 | 3 | 2 | 7 | Moderate–High |
| Al-harethy et al., 2017, Saudi Arabia | 2 | 3 | 2 | 7 | Moderate–High |
| Rhee et al., 2017, South Korea | 2 | 3 | 2 | 7 | Moderate–High |
| Sinno et al., 2014, Canada | 2 | 3 | 2 | 7 | Moderate–High |
| Ahmed et al., 2014, USA | 2 | 4 | 2 | 8 | High |
| Brown et al., 2013, USA | 1 | 4 | 3 | 8 | High |
| Broer et al., 2012, USA | 2 | 4 | 2 | 8 | High |
| Armijo et al., 2012, USA | 1 | 4 | 2 | 7 | Moderate–High |
| McArdle et al., 2012, UK | 1 | 3 | 2 | 6 | Moderate |
| Gomes et al., 2011, Brazil | 2 | 2 | 2 | 6 | Moderate |
| Biller et al., 2009, USA | 1 | 4 | 2 | 7 | Moderate–High |
| Dobke et al., 2006. USA | 2 | 3 | 2 | 7 | Moderate–High |
| Choe et al., 2004, USA | 2 | 2 | 2 | 6 | Moderate |
| Pearson et al., 2004 | 2 | 4 | 2 | 8 | High |
