# Supplementary material for: Nasal Aesthetic Preferences Across Populations: A Systematic Review of Cultural, Ethnic, and Generational Trends
Source: Aesthet Surg J. 2026 Mar 27;46(8):NP73–80. doi: 10.1093/asj/sjag062 (PMC13458594; doi:10.1093/asj/sjag062)
Supplement: Supplementary file 1 [file sjag062_supplementary_data.zip › Appendix E.docx]

**Appendix E.** Summary of Methodological Quality Appraisal Using the AXIS Framework

| Study (Author, Year) | Introduction (Max 1) | Methods (Max 10) | Results (Max 5) | Discussion (max 2) | Other (max 2) | Overall Score (out of 20) | Overall Recommendation |
| --- | --- | --- | --- | --- | --- | --- | --- |
| Okumura et al., 2025 | 1 | 8 | 5 | 2 | 2 | 18 | High Quality |
| Torbey et al., 2024 | 1 | 8 | 5 | 2 | 2 | 18 | High Quality |
| Liu et al., 2024 | 1 | 8 | 5 | 2 | 2 | 18 | High Quality |
| Al-Shawaf et al., 2024 | 1 | 8 | 5 | 2 | 2 | 18 | High Quality |
| Patel et al., 2023, USA | 1 | 7 | 5 | 2 | 2 | 17 | High Quality |
| Tavakoli et al., 2023, Iran | 1 | 8 | 5 | 2 | 2 | 18 | High Quality |
| Frederick et al., 2022, USA | 1 | 8 | 5 | 2 | 2 | 18 | High Quality |
| Sun et al., 2022, China | 1 | 8 | 5 | 2 | 2 | 18 | High Quality |
| Liang et al., 2022, China | 1 | 8 | 5 | 2 | 2 | 18 | High Quality |
| Corduff et al., 2020, Singapore | 1 | 8 | 5 | 2 | 2 | 18 | High Quality |
| Parab et al., 2019, India | 1 | 8 | 5 | 2 | 2 | 18 | High Quality |
| Balaji et al., 2019, India | 1 | 8 | 5 | 2 | 2 | 18 | High Quality |
| Samizadeh et al., 2018, China | 1 | 9 | 5 | 2 | 2 | 19 | High Quality |
| Samizadeh et al., 2018, China | 1 | 9 | 5 | 2 | 2 | 19 | High Quality |
| Mafi et al., 2018, Iran | 1 | 7 | 5 | 2 | 2 | 17 | High Quality |
| Saad et al., 2018, Spain | 1 | 8 | 5 | 2 | 2 | 18 | High Quality |
| Al-harethy et al., 2017, Saudi Arabia | 1 | 7 | 5 | 2 | 2 | 17 | High Quality |
| Rhee et al., 2017, South Korea | 1 | 8 | 5 | 2 | 2 | 18 | High Quality |
| Sinno et al., 2014, Canada | 1 | 8 | 5 | 2 | 2 | 18 | High Quality |
| Ahmed et al., 2014, USA | 1 | 8 | 5 | 2 | 2 | 18 | High Quality |
| Brown et al., 2013, USA | 1 | 8 | 5 | 2 | 2 | 18 | High Quality |
| Broer et al., 2012, USA | 1 | 8 | 5 | 2 | 2 | 18 | High Quality |
| Armijo et al., 2012, USA | 1 | 8 | 5 | 2 | 2 | 18 | High Quality |
| McArdle et al., 2012, UK | 1 | 7 | 5 | 2 | 2 | 17 | High Quality |
| Gomes et al., 2011, Brazil | 1 | 7 | 5 | 2 | 2 | 17 | High Quality |
| Biller et al., 2009, USA | 1 | 7 | 5 | 2 | 2 | 17 | High Quality |
| Dobke et al., 2006. USA | 1 | 7 | 5 | 2 | 2 | 17 | High Quality |
| Choe et al., 2004, USA | 1 | 8 | 5 | 2 | 2 | 18 | High Quality |
| Pearson et al., 2004 | 1 | 7 | 5 | 2 | 2 | 17 | High Quality |
